# Effects of foot progression angle on kinematics and kinetics of a cutting movement

**DOI:** 10.1186/s40634-022-00447-1

**Published:** 2022-01-18

**Authors:** Kohei Nishizawa, Takeshi Hashimoto, Satoshi Hakukawa, Takeo Nagura, Toshiro Otani, Kengo Harato

**Affiliations:** 1 Graduate School of Health Management, Keio University, 4411 Endo, Fujisawa, Kanagawa 252-0883 Japan; 2 Sports Medicine Research Center, Keio University, Yokohama, Kanagawa Japan; 3 Department of Orthopedic Surgery, Keio University School of Medicine, Shinjuku, Tokyo Japan; 4 Department of Clinical Biomechanics, Keio University School of Medicine, Shinjuku, Tokyo Japan; 5 International University of Health and Welfare Ichikawa Hospital, Ichikawa, Chiba Japan

**Keywords:** Anterior cruciate ligament injuries, Knee joint, Biomechanics, Movement

## Abstract

**Purpose:**

Foot progression angle is a key factor for biomechanical knee load, which is associated with noncontact anterior cruciate ligament (ACL) injury during sports-specific tasks. The purpose of the present study was to assess the biomechanics of trunk, pelvis, and lower extremities during a cutting maneuver under different foot progression angles.

**Methods:**

Nineteen male collegiate athletes (ages 18–24) participated in the present study. Cutting motion was analyzed using eight infrared cameras (250 Hz), two force plates (1250 Hz), and 44 reflective markers. Subjects performed 45-degree side cutting maneuvers under three foot progression angles, including 20 degrees (toe-out: TO), 0 degrees (neutral: TN), and − 20 degrees (toe-in: TI). Peak values of each biomechanical parameters in trunk, pelvis, hip, and knee within a first 40% stance phase and each parameter at the timing of the peak vertical ground reaction force were assessed. A statistical analysis was performed to compare data among the three-foot progression angles using the Friedman test.

**Results:**

Peak angles of knee abduction, tibial internal rotation, hip internal rotation, and hip adduction were significantly greater for TI position than for TO position (*p* < 0.01). Peak moments of knee abduction and tibial internal rotation under TI position were also significantly larger than TO position (*p* < 0.01). Moreover, greater peak pelvis-trunk rotation was found for TI position than for TN and TO positions (*p* < 0.01).

**Conclusion:**

From the present study, TI position could lead to an increased risk of ACL injury during a pre-planned cut maneuver, compared to TO position.

## Background

Noncontact anterior cruciate ligament (ACL) injury in athletes is commonly seen during deceleration, side-cutting, or landing tasks in various sports activities [15, 22, 25]. Knee valgus is an important biomechanical consideration for injury mechanism, based on previous three-dimensional biomechanical analysis [3, 11, 20, 22, 26, 28, 29]. For instance, a video imaging analysis reported that athletes exhibited knee valgus, extension, and an external rotation position at the time of several injury situations [5, 15]. In addition, an increased knee abduction angle during a drop landing task was characterized as a risk factor of noncontact ACL injury [28]. A prospective study assessed the kinetic parameters of a jump-landing task at baseline, and athletes who had sustained an ACL injury exhibited a 2.5-times knee valgus moment, compared with those who had an intact ACL [11]. ACL injured athletes showed greater knee internal rotation angle during cutting motion at baseline than control [39]. Prevention programs for ACL injury have focused on reducing knee valgus loading during sporting activities [6, 7, 12, 21]. The training program regarding modification of trunk motion and foot landing position was effective for the reduction of knee valgus loading during cutting motion [6]. Therefore, trunk, hip, and foot motion during sports movement are important as well as knee joint motion for ACL injury prevention [2, 9, 14, 24, 34, 35].

A cutting maneuver is a high-demand sports activity that has been recognized as a risky movement associated with noncontact ACL injuries [29]. According to previous reports, the cutting maneuvers produces an increased mechanical load at the knee joint, compared with hopping and drop landing [27, 36]. Foot progression angle at initial contact is also known to affect knee biomechanics during high demand activities. In term of cutting maneuver, Sigward et al. found that athletes with large knee valgus moment indicated increased internally rotation foot angle compared to those with normal knee valgus moment [33]. Jones et al. found that athletes with a larger knee valgus moment presented a more internally rotated foot progression angle than those with a smaller mean knee valgus moment [16]. Therefore, the foot progression angle is a key factor for the knee joint load during a cutting movements, and toe-in landing seems to be related to an increased knee valgus loading which is known as an ACL injury risk factor [16, 33]. Clinically, the kinematic chain mechanism of whole-body segments is extremely important to consider ACL injury prevention programs. However, few studies have been done to compare biomechanical differences, including trunk, pelvis, and lower extremities, in cutting maneuvers among different foot progression angles. Specifically, no previous study compared the biomechanical parameters between toe-in and toe-out positions during a cutting movement.

The purpose of the present study was to investigate and clarify whether difference of foot progression angles would affect the biomechanics of the trunk, pelvis, and lower extremities during a cutting maneuver. It was hypothesized that foot progression angles would change movements of trunk and pelvis as well as biomechanics of the hip and knee joints, and toe-in position could indicate the highest risk of noncontact ACL injury.

## Methods

### Study type

Cross-sectional study was conducted to investigate whether foot progression angle would affect biomechanical parameters of knee, hip, pelvis, and trunk during a cutting movement.

### Subjects

A total of 16 subjects were needed to detect a significant difference of 3.5 degrees (standard deviation [SD] 6) for the knee abduction angle (β = 0.80, α = 0.05) based on power analysis of previous research examining the effects of toe angle [30]. In the present study, nineteen male collegiate athletes with a mean age of 20 ± 1.5 years, a mean height of 175 ± 5.4 cm, and a mean weight of 67 ± 6.5 kg were enrolled. Age of the subjects ranged from 18 to 24 years. The mean Tegner activity level scale was 7.0 ± 0.2 level, and the subjects in the current investigation consisted of twelve soccer players, five ice hockey players, one handball player, and one Judo player. The subjects were recruited in university sports club, and the inclusion criteria was more than Tegner activity score level 6. None of the subjects had a severe musculoskeletal injury in the trunk or lower extremities requiring surgery. All the subjects signed a written informed consent approved by university institutional review board.

### Instrumentation

Three-dimensional motion analysis was performed using two ground reaction force (GRF) plates (sampling frequency, 1250 Hz; AM6110, Bertec, Columbus, OH, USA), eight infrared cameras (250 frames/s; Miqus, Qualisys, Sweden) and 44 retro-reflective markers. The GRF data was synchronized with the motion capture data. To define the local coordinate system for each segment, anatomical landmark markers were placed on bilateral acromion processes, xiphoid process, suprasternal notch, 7th cervical vertebra, 10th thoracic vertebra, bilateral anterior/posterior superior iliac spine, bilateral greater trochanters, bilateral lateral/medial epicondyles, bilateral lateral/medial malleoli, bilateral posterior heels, bilateral navicular bone, and bilateral heads of the first/fifth metatarsals. Four tracking markers were placed in squares on the anterior aspect of the thigh and shank.

### Procedures

**Fig. 1:**
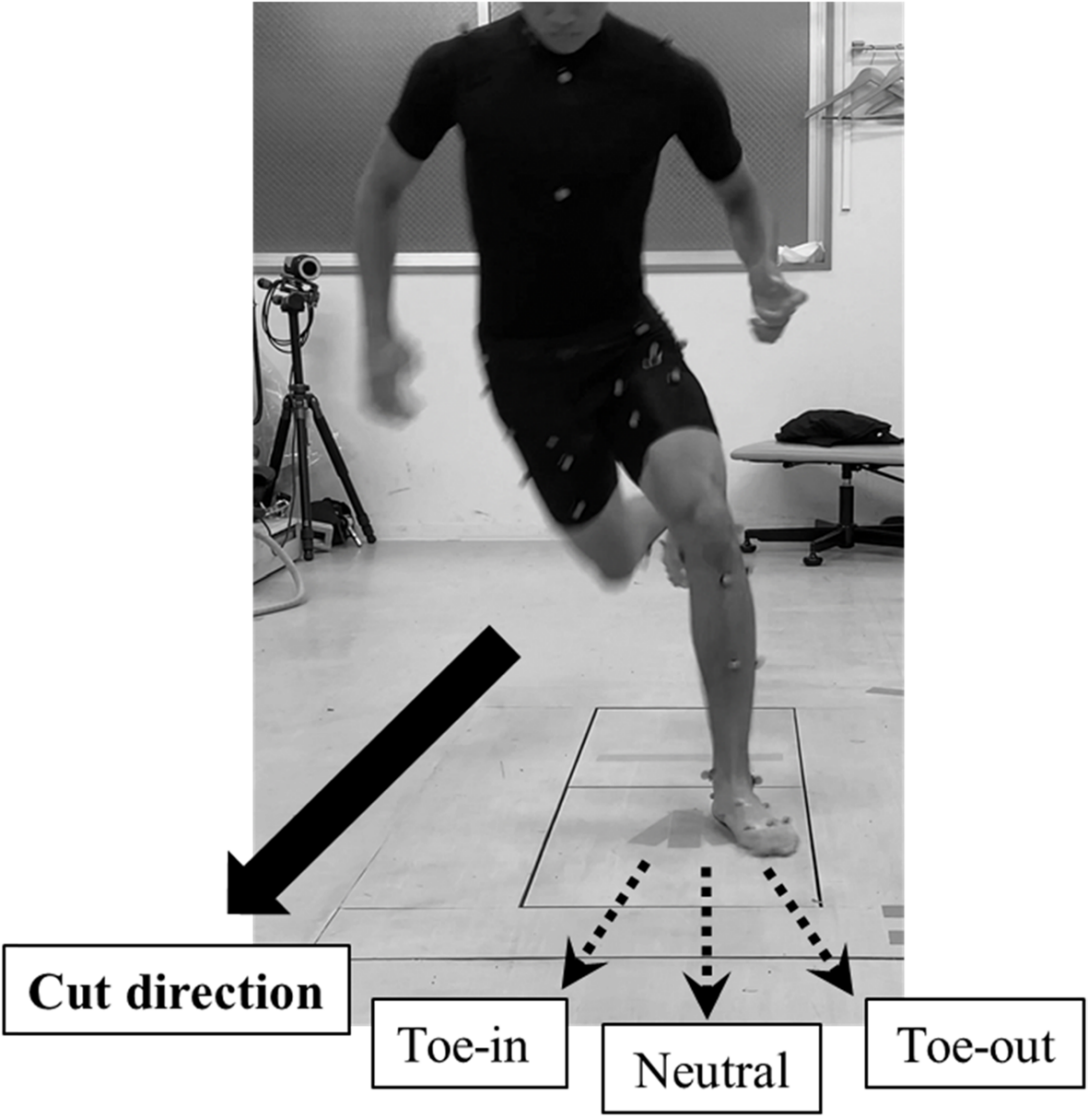
Participants perform cutting maneuver on nondominant leg under toe-in, neutral, and toe-out landing. The peak values within the 40% stance phase equivalent to the loading acceptance and value at the timing of the peak Fz were compared among the neutral, toe-out, and toe-in positions

The participants were asked to sprint as fast as they can and then performed a 45-degree side cutting task to the side opposite to the tested limb at a self-selected speed with bare feet. In the present protocol, the cutting movement was done under three different foot progression angles, including 0 degrees (toe-neutral [TN]),− 20 degrees (toe-in [TI]), and 20 degrees (toe-out [TO]). The subjects were asked to aim their toe according to the signs attached to the force plate (Fig. 1). After participants performed the jogging and 45-degree side cutting task several times as a warm-up, two successful motion data sets were obtained for each pattern. The nondominant leg (1 right, 18 left) was chosen for the measurement. The dominant leg was defined as the leg with which each athlete preferred to kick a ball [1, 10].

### Data analysis

The three-dimensional kinematics of trunk, pelvis, hip and knee, and kinetics of hip and knee were calculated using Visual 3D (C-Motion Company, Rockville, MD). A low-pass filter was used to smooth the marker and GRF data at a cutoff frequency of 12 Hz, based on previous study [21]. Each parameter was normalized from zero to 100 between the initial contact and the toe off. External joint moments were calculated using inverse dynamics. The laboratory coordinate system was set to the right of progression direction as X-axis, progression direction as Y-axis, and vertical direction as Z-axis. The angle of the foot coordinate system with respect to the Z-axis of the laboratory coordinate system was defined as the toe angle (inward rotation: negative, outward rotation: positive). A positive value of pelvis lateral tilt was defined as an inclination toward the dominant leg side in the coronal plane. A positive value of trunk lateral bending was defined as an inclination toward the non-dominant leg side in the coronal plane. Pelvic and trunk rotations were evaluated as the angle relative to the jogging direction, and positive values were defined as rotation toward the nondominant leg side (measured side). The pelvis-trunk angle was calculated by the trunk motion relative to the pelvis. The peak values of the angle and moment within 40% after initial contact and at the timing of the peak vertical ground reaction force (vGRF) during the stance phase were analyzed as the weight acceptance phase, which is known to be a risky timing for noncontact ACL injury [4].

### Statistical analysis

After Shapiro–Wilk test was performed to confirm normality assumption, the Friedman test was used to compare the differences among TI, TN and TO positions during a cutting movement using SPSS Statistics for Windows, Version 23.0 (IBM Corp., New York, USA). The average value of two successful trials for each foot progression angle was used in the statistical analysis. *P* values of < 0.05 were regarded as indicating significant differences. As a post-hoc test, the Wilcoxon signed-rank test was corrected using the Bonferroni method to compare the differences between foot progression angle patterns. Effect size was the difference of kinematics and kinetics between foot progression angle patterns, obtained from Wilcoxon signed-rank test.

## Results

The toe angles at initial contact were − 12.6 (4.7) degrees, 2.3 (5.6) degrees and 19.5 (8.1) degrees in the TI, TN and TO positions, respectively. Significant differences were found among the three-foot progression angles (*p* < 0.001). The peak value of each parameter during the 40% stance phase is shown in Table 1. Peak knee abduction, internal rotation, hip adduction, internal rotation, and pelvis-trunk rotation angle were significantly greater for TI position than for TO position (*p* < 0.01). Peak pelvis-trunk flexion angle was smaller for TI position than TO position (*p* = 0.016). Peak knee abduction and internal rotation moment under TI position was larger than TO position (*p* < 0.01). Each biomechanical parameter at the timing of the peak Fz is shown in Table 2. TI position resulted in a significantly greater knee abduction, internal rotation, hip internal, rotation pelvis-trunk rotation angle than TO position (*p* < 0.01).

**Table 1:** Mean (SD) value of peak kinematic and kinetic parameters within a 40% stance phase during the cutting maneuver

	TI	TN	TO	*P* Value^*^	Effect size r^d^		
					TI-TN	TI-TO	TN-TO
**Kinematic parameters (deg.)**							
**Knee**							
Flexion angle (Flexion: + ; Extension: -)	52.0 (9.3)	51.2 (8.6)	53.2 (7.4)	n.s	0.17	0.24	0.37
Abduction angle (Abduction + ; Adduction: -)	2.3 (7.1)	-0.1 (5.2)	-3.1 (5.9)	< .001	0.51	0.80^b^	0.76^c^
Internal rotation angle (Internal: + ; External: -)	0.4 (7.5)	-1.5 (7.6)	-3.7 (10.0)	< .05	0.56^a^	0.75^b^	0.52
**Hip**							
Flexion angle (Flexion: + ; Extension: -)	45.2 (16.5)	46.5 (15.1)	49.0 (14.9)	n.s	0.15	0.63^b^	0.54
Adduction angle (Adduction + ; Abduction: -)	0.9 (5.4)	-1.7 (5.0)	-3.4 (5.1)	< .05	0.38	0.61^b^	0.44
Internal rotation angle (Internal: + ; External: -)	6.2 (10.9)	2.8 (9.8)	-0.1 (9.3)	< .001	0.60^a^	0.83^b^	0.67^c^
**Pelvis-trunk**							
Flexion angle (Flexion: + ; Extension: -)	17.8 (10.8)	19.9 (10.2)	20.5 (11.7)	< .05	0.45	0.55^b^	0.06
Lateral bending angle (Nondominant: + ; Dominant: -)	21.0 (6.3)	20.0 (6.3)	19.6 (6.2)	n.s	0.18	0.21	0.06
Rotation angle (Nondominant: + ; Dominant: -)	20.4 (10.0)	14.2 (7.5)	10.8 (7.6)	< .001	0.66	0.83^b^	0.53
**Kinetic parameters (Nm per kg)**							
**Knee**							
Flexion moment (Flexion: + ; Extension: -)	3.0 (0.7)	2.8 (0.6)	3.0 (0.6)	n.s	0.42	0.01	0.30
Abduction moment (Abduction + ; Adduction: -)	1.3 (0.9)	0.7 (0.4)	0.6 (0.5)	< .05	0.57^a^	0.66^b^	0.22
Internal rotation moment (Internal: + ; External: -)	0.8 (0.5)	0.6 (0.5)	0.4 (0.4)	< .05	0.28	0.66^b^	0.30
**Hip**							
Flexion moment (Flexion: + ; Extension: -)	6.4 (3.5)	5.9 (3.3)	6.2 (3.8)	n.s	0.07	0.07	0.10
Adduction moment (Adduction + ; Abduction: -)	2.2 (0.7)	2.0 (0.6)	2.3 (0.8)	n.s	0.13	0.15	0.25
Internal rotation moment (Internal: + ; External: -)	1.7 (1.2)	1.6 (0.9)	2.0 (1.4)	n.s	0.08	0.20	0.46

^*^Values determined using the Friedman test

^a^Significant difference between Toe-in (TI) and Toe-neutral (TN) positions

^b^Significant difference between TI land Toe-out (TO) positions

^c^Significant difference between TN and TO positions

^d^Effect size was calculated using Wilcoxon signed-rank test

**Table 2:** Mean (SD) value of kinematic and kinetic parameters at peak Fz during cutting maneuver

	TI	TN	TO	*P* Value^*^	Effect size^d^		
**Kinematic parameters (deg.)**					TI-TN	TI-TO	TN-TO
**Knee**							
Flexion angle (Flexion: + ; Extension: -)	35.6 (9.4)	35.9 (12.2)	35.5 (10.5)	n.s	0.04	0.06	0.09
Abduction angle (Abduction + ; Adduction: -)	0.8 (7.1)	-1.9 (5.0)	-5.2 (5.6)	< .001	0.63^a^	0.85^b^	0.81^c^
Internal rotation angle (Internal: + ; External: -)	-5.9 (10.1)	-8.2 (8.1)	-12.2 (11.5)	< .05	0.26	0.72^b^	0.49
**Hip**							
Flexion angle (Flexion: + ; Extension: -)	39.3 (19.3)	41.1 (16.1)	44.0 (16.7)	< .05	0.16	0.71^b^	0.43
Adduction angle (Adduction + ; Abduction: -)	-2.5 (5.9)	-4.6 (4.7)	-5.5 (6.2)	n.s	0.30	0.39	0.18
Internal rotation angle (Internal: + ; External: -)	0.7 (12.8)	-1.9 (10.1)	-5.2 (10.2)	< .05	0.28	0.71^b^	0.58^c^
**Pelvis-trunk**							
Flexion angle (Flexion: + ; Extension: -)	10.8 (12.4)	12.8 (11.3)	12.3 (13.0)	n.s	0.23	0.29	0.11
Lateral bending angle (Nondominant: + ; Dominant: -)	17.2 (6.1)	15.9 (6.0)	15.7 (6.9)	n.s	0.19	0.17	0.00
Rotation angle (Nondominant: + ; Dominant: -)	15.7 (10.6)	9.2 (8.1)	5.7 (7.3)	< .001	0.49	0.87^b^	0.49
**Kinetic parameters (Nm per kg)**							
**Knee**							
Flexion moment (Flexion: + ; Extension: -)	1.5 (1.6)	1.0 (1.5)	0.7 (2.0)	n.s	0.31	0.49	0.23
Abduction moment (Abduction + ; Adduction: -)	0.6 (1.2)	-0.6 (0.8)	-1.1 (0.9)	< .001	0.74^a^	0.88^b^	0.37
Internal rotation moment (Internal: + ; External: -)	0.1 (0.7)	-0.4 (0.4)	-0.6 (0.4)	< .05	0.64^a^	0.72^b^	0.47
**Hip**							
Flexion moment (Flexion: + ; Extension: -)	4.1 (4.4)	5.2 (4.8)	5.5 (5.2)	n.s	0.28	0.42	0.15
Adduction moment (Adduction + ; Abduction: -)	-0.03 (1.9)	0.9 (0.9)	1.2 (1.3)	n.s	0.46	0.38	0.07
Internal rotation moment (Internal: + ; External: -)	0.3 (1.2)	1.2 (1.2)	1.1 (1.2)	< .001	0.70^a^	0.53	0.03

^*^Values determined using the Friedman test

^a^Significant difference between Toe-in (TI) and Toe-neutral (TN) positions

^b^Significant difference between TI land Toe-out (TO) positions

^c^Significant difference between TN and TO position

^d^Effect size was calculated using Wilcoxon signed-rank test

**Fig. 2:**
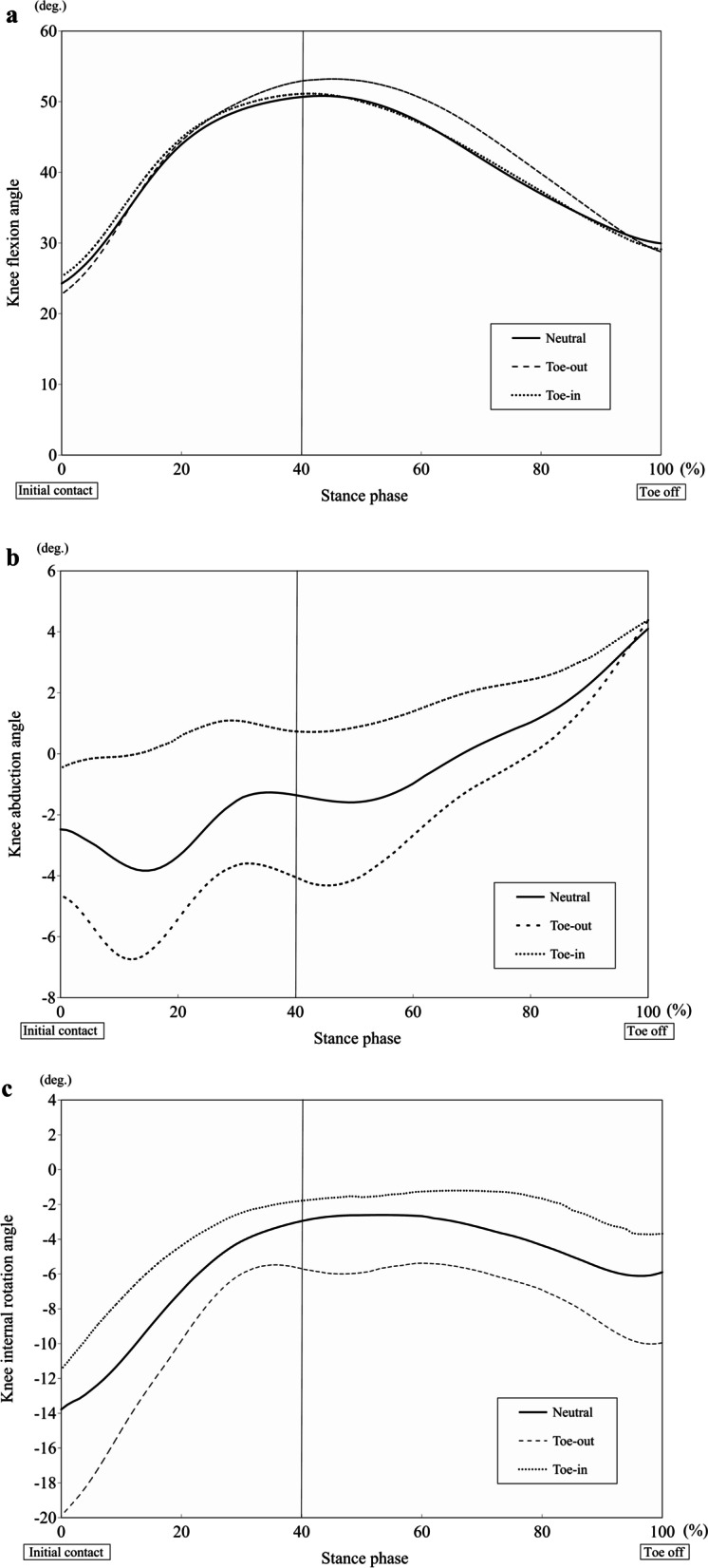
Waveforms showing the average knee flexion angle (**a**), knee abduction angle (**b**), and knee internal rotation angle (**c**) during the stance phase of the cutting maneuver

**Fig. 3:**
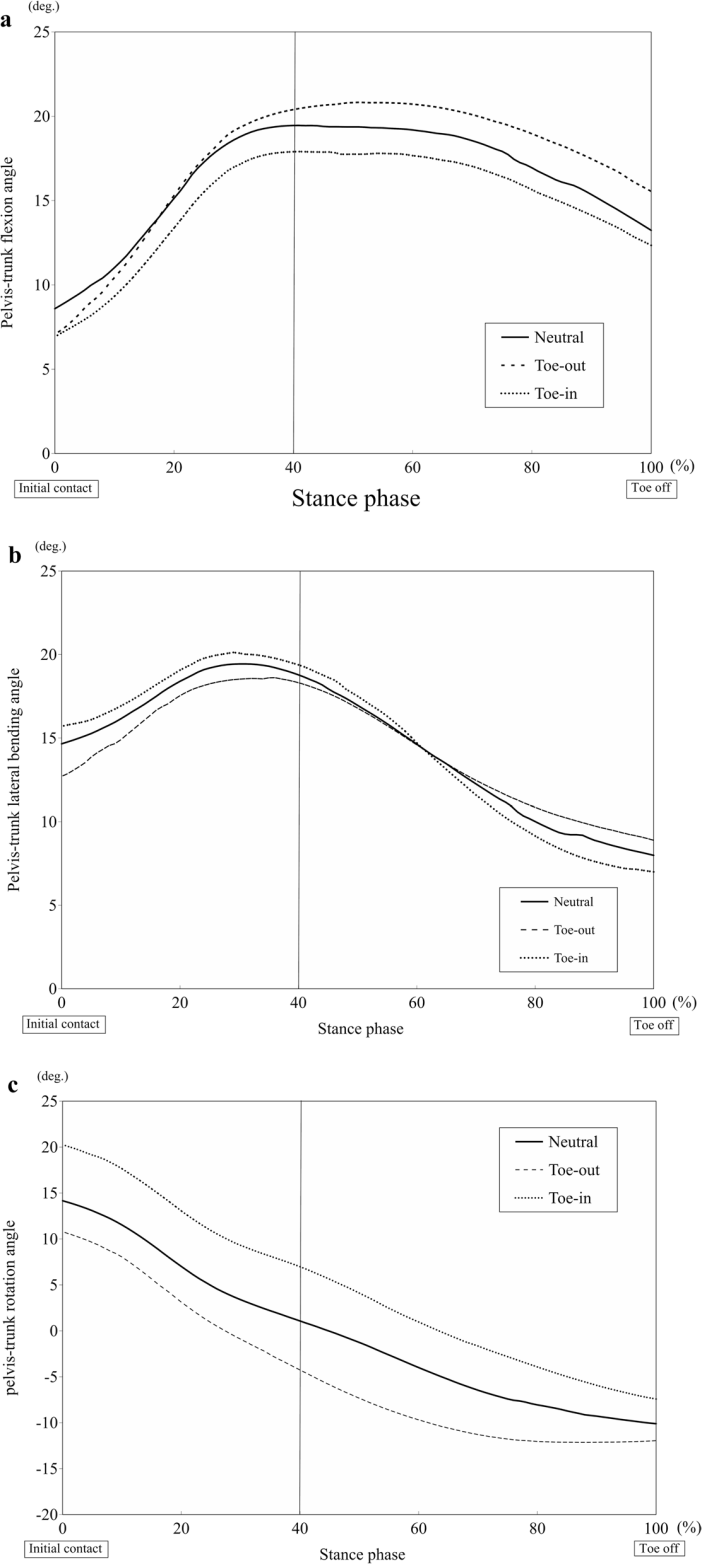
Waveforms showing the average pelvis-trunk flexion angle (**a**), pelvis-trunk lateral bending angle (**b**), and pelvis-trunk rotation angle (**c**) during the stance phase of the cutting maneuver

**Fig. 4:**
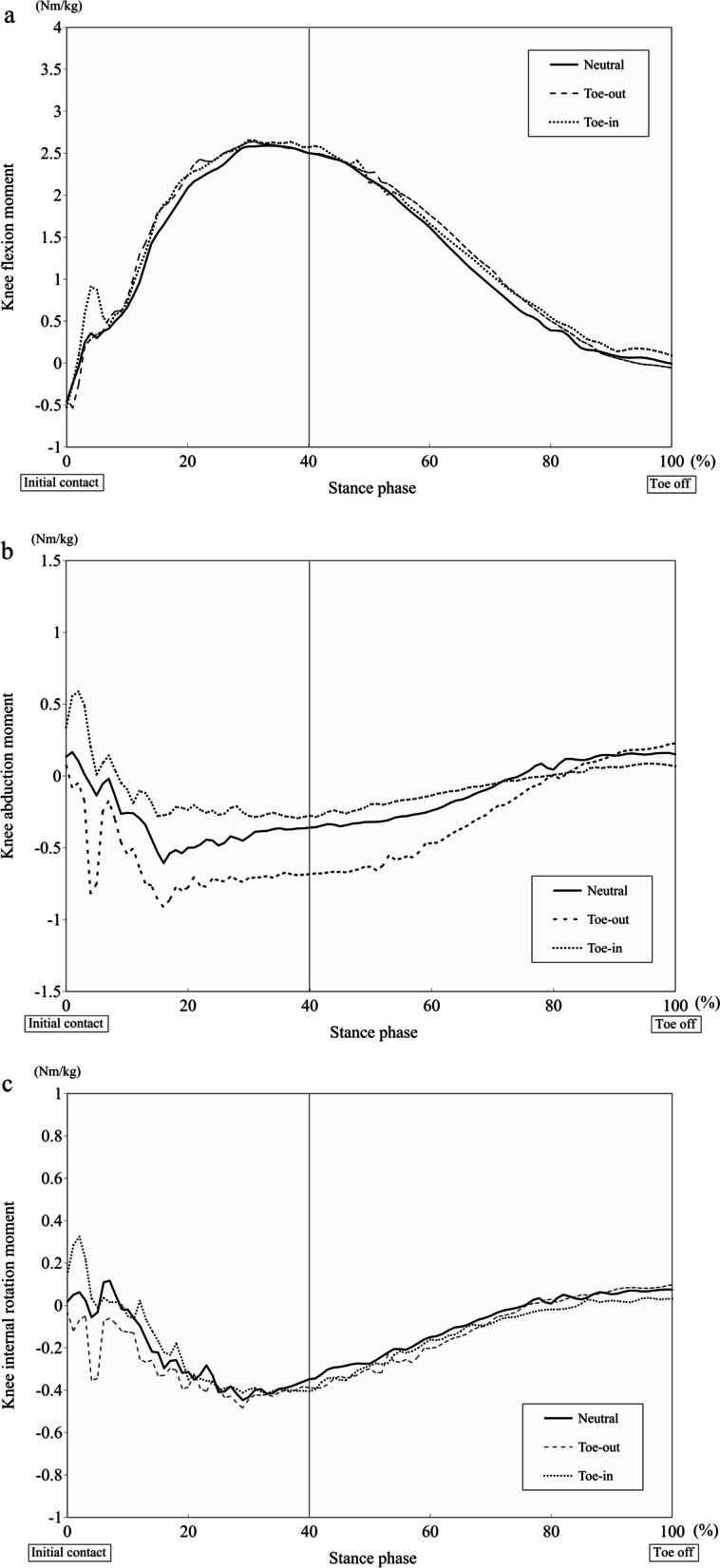
Waveforms showing the average knee flexion moment (**a**), knee abduction moment (**b**), and knee internal rotation moment (**c**) during the stance phase of the cutting maneuver

The three-dimensional kinematic waveforms of the knee joints and trunk-pelvis during the stance phase are shown in Figs. 2 and 3, and kinetic waveform of knee joint during the stance phase is shown in Fig. 4.

## Discussion

The results of the current investigation supported the hypothesis that the foot progression angle would change the biomechanics of the trunk and pelvis, as well as the lower extremities. The main finding of the present study was that larger angles and moments of knee valgus and tibial internal rotation were observed for TI position, with biomechanical changes in the hip joint, trunk, and pelvis. Specifically, the kinematic chain mechanism including the knee, hip, pelvis, and trunk was affected by three different foot progression angles during the weight acceptance phase. The present study suggested that larger trunk torsion was related to larger knee abduction and tibial internal rotation with hip adduction and internal rotation under an internally rotated foot progression angle during a cutting movement, which was considered as risky movement of noncontact ACL injury [17, 18, 23, 31]. To our knowledge, this is the first study to assess differences in the cutting maneuver according to different foot progression angles.

Previous studies have investigated the relationship between the foot rotation angle and knee valgus loading during sports-specific tasks [13, 16, 21, 30, 33, 37, 38]. Sigward et al. found that the athletes with a high knee valgus moment produced a more internally rotated foot progression angle, compared with those with a normal knee valgus moment, during a cutting maneuver [33]. However, they showed no difference in the knee abduction angle between the subjects with and without high knee valgus moment. Unlike previous protocol, the foot progression angle was set at TI, TN, and TO with a difference 20 degree each in the current study, and the large difference between TI, TN, and TO might affect knee abduction motion during a cutting maneuver. In addition, preplanned foot progression angle in a jump-landing task was associated with knee valgus loading, and TI landing led to a larger knee valgus angle and moment than TO landing [13, 30]. These findings were consistent with the present study, since TI direction resulted in a larger knee abduction angle and moment than TN and TO directions in both parameters. The current results showed that TI remained in a knee abduction position during the 40% stance phase, while a knee adduction movement was observed during the early weight acceptance phase (0%-20%) in the TN and TO positions (Fig. 2). Knee abduction movement was associated with knee valgus moment during a cutting movement, therefore, the lack of knee adduction movement may contribute to the increase in the knee valgus moment during the weight acceptance phase (Fig. 4) [16, 21].

On the other hand, Kristianslund et al. indicated that the foot progression angle was not associated with the knee abduction moment during handball offensive cut [21]. Contrary to the current study, the previous protocol involved the subjects conducting a cutting maneuver in front of a virtual defender while holding a ball, which resulted in greater hip external rotation [21]. Presumably, differences in the trunk or hip joint movement patterns may affect the knee abduction moment. According to a previous study, the hip internal rotation angle during a cutting motion is a key predictor of the knee valgus moment [24, 32, 33]. In addition, hip adduction angle was associated with knee valgus moment [21]. In current results, greater peak hip adduction and internal rotation angle were shown in TI position than in TO position. Therefore, hip internal rotation and adduction with an internally rotated foot is a kinematic chain mechanism for knee abduction, and neuromuscular control training at the hip joint may reduce the knee valgus loading, which is considered as a risk of ACL injury.

An increased trunk torsion relative to the pelvis occurred for the TI position as a result of the compensation movements associated with the different foot progression angles. Simulated cutting movement with greater trunk rotation increased the knee internal rotation moment [7]. In addition, using a specimen model in a simulated jump-landing, the coupling moment of knee abduction and internal rotation resulted in a greater ACL strain, compared with that observed in individuals [17, 18, 23, 31]. Knee abduction and the internal rotation moment in both parameters increased for TI position than for TO position in the present study. Therefore, a cutting motion under TI direction was regarded as the ACL injury risk position, compared with TN and TO directions. The video analysis assessed the biomechanics at the ACL injury scenes of football games and showed that trunk torsion was increased, but the foot position was externally rotated [5]. Whereas inward foot rotation motion was observed in the injury situations of handball and basketball games [19]. Therefore, the results of the TI position more simulated injury situations with preplanned cut in handball and basketball.

The present study found that TI position led to greater knee abduction, hip internal rotation, and smaller trunk flexion as compared with TO position during cutting motion. The cutting movement assessment score (CMAS) shows that the pattern in this study increases the risk of ACL injury [8]. Therefore, the instruction to avoid TI position during cutting maneuver in the clinical situation by therapists and trainers is effective for ACL injury prevention.

Several limitations of the present study should be mentioned. First, all the subjects were male collegiate athletes. Generally, female athletes have a high noncontact ACL injury risk, compared with male athletes. Results obtained from female athletes may be different from the present results. Second, a cutting maneuver was assessed in different sport players. Thus, the influence of sports specificity on the present results is unknown. Third, balance and coordination abilities of athletes were not assessed prior to perform motion analysis in the current investigation. These abilities may affect kinematic and kinetic parameters during a cutting maneuver. Forth, preplanned cut motions under different foot progression angles were analyzed in the present study. The cutting motion was a simulation based on the limited movement freedom and thus may differ slightly from actual movement in games. Lastly, the actual TI angle was less than the preplanned angle. A 20-degree TI landing might be too large for athletes to perform during a cutting maneuver. However, the results of the current study provide valuable information when considering the influence of the foot progression angle on the kinematic chain that occurs during the cutting maneuver.

## Conclusion

The kinematic chain mechanism was affected including the knee, hip, pelvis, and trunk by three different foot progression angles during a cutting movement. Significantly larger angles and moments of knee abduction and tibial internal rotation with biomechanical changes were observed for TI position, compared to TN and TO positions. Therefore, athletes should be aware of their foot progression angle during cutting movements to avoid ACL injury.

## Data Availability

The datasets used and/or analyzed during the current study are available from the corresponding author on reasonable request.

## Abbreviations

ACL: Anterior cruciate ligament
TO: Toe-out
TN: Neutral
TI: Toe-in
vGRF: Vertical ground reaction force
GRF: Ground reaction force
